# Leucine-Enriched Essential Amino Acids Augment Mixed Protein Synthesis, But Not Collagen Protein Synthesis, in Rat Skeletal Muscle after Downhill Running

**DOI:** 10.3390/nu8070399

**Published:** 2016-06-28

**Authors:** Hiroyuki Kato, Hiromi Suzuki, Yoshiko Inoue, Katsuya Suzuki, Hisamine Kobayashi

**Affiliations:** Frontier Research Laboratories, Institute for Innovation, Ajinomoto Co., Inc., Kawasaki, Kanagawa 210-8681, Japan; hiromi_suzuki@ajinomoto.com (H.S.); yoshiko_inoue@ajinomoto.com (Y.I.); katsuya_suzuki@ajinomoto.com (K.S.); hisamine_kobayashi@ajinomoto.com (H.K.)

**Keywords:** muscle collagen protein synthesis, mixed muscle protein synthesis, Leucine-enriched essential amino acids, downhill running, mammalian target of rapamycin

## Abstract

Mixed and collagen protein synthesis is elevated for as many as 3 days following exercise. Immediately after exercise, enhanced amino acid availability increases synthesis of mixed muscle protein, but not muscle collagen protein. However, the potential for synergic effects of amino acid ingestion with exercise on both mixed and collagen protein synthesis remains unclear. We investigated muscle collagen protein synthesis in rats following post-exercise ingestion of leucine-enriched essential amino acids. We determined fractional protein synthesis rates (FSR) at different time points following exercise. Mixed protein and collagen protein FSRs in skeletal muscle were determined by measuring protein-bound enrichments of hydroxyproline and proline, and by measuring the intracellular enrichment of proline, using injections of flooding d_3_-proline doses. A leucine-enriched mixture of essential amino acids (or distilled water as a control) was administrated 30 min or 1 day post-exercise. The collagen protein synthesis in the vastus lateralis was elevated for 2 days after exercise. Although amino acid administration did not increase muscle collagen protein synthesis, it did lead to augmented mixed muscle protein synthesis 1 day following exercise. Thus, contrary to the regulation of mixed muscle protein synthesis, muscle collagen protein synthesis is not affected by amino acid availability after damage-inducing exercise.

## 1. Introduction

Collagen protein, the major protein of the extracellular matrix of skeletal muscle, has crucial roles in mechanical strength, and transmission of forces generated by muscle contractions [1]. The muscle collagen network provides a structural framework for skeletal muscle cells and should therefore grow with muscle hypertrophy. Collagen content can be increased by endurance training [2] or experimental compensatory hypertrophy [3]. Furthermore, lengthening contractions result in muscle damage that involves muscle fibers and the extracellular matrix [4]. Failure in the re-organization of the extracellular matrix after exercise leads to accumulation of connective tissue, which may interfere with tissue repair and functional recovery [5]. Thus, post-exercise re-organization of the skeletal muscle extracellular matrix is necessary for recovery from muscle damage.

Muscle collagen protein synthesis increases acutely following exercise [6,7] and remains elevated for as long as 3 days [8]; the same is true for mixed muscle protein synthesis [9,10,11]. Whereas mixed protein synthesis is increased by enhanced amino acid availability both at rest and following exercise [12,13], collagen protein synthesis in skeletal muscle is unaffected by enhanced availability of amino acids [14,15]. In addition, post-exercise meal ingestion does not induce further increases in already elevated muscle collagen protein synthesis 0.5–5 h following exercise [16]. However, muscle collagen synthesis peaks between 6 h and 1 day after exercise [8]. Therefore, questions remain as to whether the enhanced availability of amino acids might affect muscle collagen protein synthesis, particularly when post-exercise muscle collagen protein synthesis has peaked.

A leucine-enriched mixture of essential amino acids stimulates muscle protein synthesis at rest [17] and following exercise [18,19] via activation of mammalian target of rapamycin (mTOR) [20]. In addition to the effect on muscle protein synthesis, a leucine-enriched essential amino acid mixture facilitates recovery from muscle soreness [21]. Consumption of a leucine-enriched protein supplement induces gene expression related to extracellular matrix protein 30 min following exercise, and decreases the expression 4 h after exercise [22]. Therefore, we hypothesized that leucine-enriched amino acids may affect muscle collagen synthesis after exercise.

Thus, we investigated the effects of leucine-enriched amino acids on muscle collagen protein synthesis after downhill running exercise, which can increase the expression of collagen in rodents [23]. First, we examined time-dependent changes of muscle collagen protein synthesis after exercise by measuring the enrichment of d_3_-hydroxyproline in skeletal muscle after a flooding dose injection of d_3_-proline. Next, we examined whether an amino acid mixture could affect collagen protein synthesis and mixed protein synthesis in skeletal muscle immediately after exercise, and 1 day after exercise at which the mixed protein and collagen protein synthesis peaked.

## 2. Materials and Methods

This study was approved by the Institutional Animal Care and Use Committee of Ajinomoto Co., Inc. on 30 March 2011 (No. 20111210). All applicable international, national, and institutional guidelines for the care and use of animals were followed.

### 2.1. Animals

Female 7-week-old Wistar rats (Charles River Laboratories Japan, Inc., Yokohama, Japan) were housed in a temperature-controlled room on a 12-h light-dark cycle (light 10:00–22:00 and dark 22:00–10:00), and provided water and CR-F1 standard commercial chow (Charles River Laboratories Japan, Inc., Yokohama, Japan) ad libitum. After 1 week of acclimatization, rats were used for this experiment.

### 2.2. Experimental Design

Data were obtained from multiple studies. A total of 30 overnight-fasted rats were used as a sedentary control group (Sed), and a total of 64 rats underwent intermittent running on a motor-driven treadmill, at a speed of 17 m/min, for a total of 130 min on downhill (−13.5°) tracks [23]. During the exercise, 26 repetitions of five-minute running bouts were separated by 2-min intervals. The rats were adapted to the treadmill running with a lower speed for 3 days before the trial. Rats were orally administered distilled water (Con), immediately, 3 h, 1 day, 2 days, 4 days, and 7 days after the completion of the exercise, or a leucine-enriched essential amino acid mixture (1 g/kg; AminoL40) immediately and 1 day after the exercise. Mixed protein synthesis and collagen protein synthesis in skeletal muscle were determined as fractional synthesis rates (FSR, %/h) using the flooding dose method. Thirty minutes after oral administration, rats were intravenously injected with flooding doses of proline (2.8 mmol/kg) containing L-2, 5, 5-d_3_-proline (50 MPE, Sigma-Aldrich, St. Louis, MO, USA) into their tail veins. This flooding dose of proline was selected because we had previously confirmed that it increases the intracellular enrichment of proline and remains steady for 30 min following injection. A blood sample was withdrawn from abdominal aorta of each rat under inhalation anesthesia (1.5% isoflurane). Subsequently, the vastus lateralis muscle was removed, frozen in liquid nitrogen, and stored at −80 °C.

### 2.3. Leucine-Enriched Essential Amino Acids

The LEAAs mixture consisted of essential amino acids in the following proportions: histidine, 2%; isoleucine, 11%; leucine, 40%; lysine, 17%; methionine, 3%; phenylalanine, 7%; threonine, 9%; tryptophan, 1% and valine, 11%. Except for the elevated proportion of leucine, this mixture contains the ratio of essential amino acids found in whey protein; all amino acids were manufactured by Ajinomoto Co., Inc., Tokyo, Japan. The AminoL40 mixture was deliberately developed to avoid decreasing the availability of the other EAAs while increasing the proportion of leucine. In addition, the AminoL40 mixture may alleviate the impairment of muscle protein synthesis after eccentric contraction in rats [21].

### 2.4. Measurements of Blood Variables

Blood was separated from plasma by centrifugation at 10,000 rpm for 10 min at 4 °C, and the plasma was stored at −80 °C. Plasma amino acid concentrations in a part of sedentary rats and exercised groups of rats administered water (Control) or AminoL40 (1 g LEAA/kg) immediately or 1 day after exercise were measured with an automatic amino acid analyzer (JLC-500; JEOL, Tokyo, Japan).

### 2.5. Measurement of Mixed and Collagen Protein Synthesis

Contamination from non-collagen protein can lead to misinterpretations of collagen metabolism. This misinterpretation risk stems from protein synthesis rates in the protein fractions (i.e., myofibrillar proteins and sarcoplasmic proteins), which are substantially greater than those rates for collagen protein fractions [8,14,15,16]; moreover, myofibrillar protein synthesis is increased by exercise and amino acid availability [24]. To avoid this potential problem, collagen protein synthesis was calculated by measuring labeled hydroxyproline, which had been generated by the hydroxylation of injected labeled proline in skeletal muscle [25,26,27,28]. The collagen molecule undergoes posttranslational hydroxylation of proline before being extruded from the endoplasmic reticulum [4]. Hydroxyproline cannot be recycled into protein, so it should be found in only collagen protein [29]. Approximately 30 mg of the vastus lateralis muscle was homogenized in 15% sulfosalicylic acid, and the homogenate was centrifuged at 10,000 rpm for 10 min at 4 °C. The supernatant was used for measurement of the enrichment of intracellular free proline in the vastus lateralis muscle. The precipitate, which was hydrolyzed in 2 mL of 6N hydrochloric acid at 90 °C for 16 h, was used for measurement of the enrichment of protein-bound proline and hydroxyproline in the vastus lateralis muscle. Amino acids in the supernatant and the hydrolysate were purified using cation exchange chromatography (Dowex 50 W 8X; Bio-Rad Laboratories, Hercules, CA, USA) and dried in a rotary evaporator (Nakajima Corp., Tokyo, Japan). D_3_-proline enrichment (E_(Pro, muscle free)_) in the supernatant was determined by its tert-butyl dimethylsilyl derivatization (N-methyl-N-tert-butyldimethylsilytrifluoroacetamide, Thermo Fisher Scientific, Waltham, MA, USA) using gas chromatography-mass spectrometry (GC-MS; 6890 GC system and 5973 Network Mass Selective Detector, Agilent, Santa Clara, CA, USA). We used the MS to monitor ions 286.2 and 289.2 in the electron impact mode. Muscle protein-bound d_3_-proline and d_3_-hydroxyproline enrichment was determined by measuring the butyl derivatization (HCl-n-butanol (10% v/v): GL Science Inc., Tokyo, Japan) with liquid chromatography–mass spectrometry-enabled ion monitoring based on the former study with some modifications [30]. A portion of 75 µL of 3N HCl-n-butanol was added to the sample residue and incubated for 15 min at 80 °C. Following butylation, the mixture was dried in a rotary evaporator and reconstituted in 400 µL of mobile phase (0.2% acetic acid). The butylated samples were separated on a 2.1 × 150 mm × 3 μm L-Column2 (Chemicals Evaluation and Research Institute, Tokyo, Japan) using a Prominence HPLC system (Shimadzu, Kyoto, Japan). Mobile phase A was 0.2% acetic acid, and mobile phase B was 0.2% acetic acid in acetonitrile. Gradient conditions were initial = 95% A and 5% B; 2.1 min = 85% A and 15% B; 6 min = 80% A and 20% B; 9 min = 65% A and 35% B; and 12.5 min = 98% A and 2% B, followed by 5 min equilibration with initial mobile phase (95% A and 5% B). MS/MS analysis was carried out using an API 3200 Triple quadrupole mass spectrometry system (SCIEX, Framingham, MA, USA). Mobile phase was introduced into the mass spectrometer via the electrospray ionization source operating in the positive ion mode at 5500 V, curtain gas at 20 psi, collision gas at 6 psi, ion source gas1 at 60 psi, and ion source gas2 at 70 psi. We monitored ions 174.2, 175.2 (for proline), 190.1, and 191.1 (for hydroxyproline) with the first mass spectrometer; we monitored ions 72.1, 72.3 (for proline), 88.2, and 89.2 (for hydroxyproline) with the second mass spectrometer; these analytical procedures used the external standard curve approach [31]. The FSR of muscle protein was calculated using the precursor–product model as described below,

Mixed protein FSR (%/h) = *E*_(Pro, protein-bound)_/(*E*_(Pro, muscle free)_ × *t*) × 100 (1)

Collagen protein FSR (%/h) = *E*_(HydroPro, protein-bound)_/(*E*_(Pro, muscle free)_ × *t*) × 100 (2)

In Equations (1) and (2), *t* represents the time interval between d_3_-proline injection and tissue sampling; *E*_(Pro, muscle free)_ represents the enrichment of precursor (intracellular proline); and *E*_(HydroPro, protein-bound)_ and *E*_(Pro, protein-bound)_ represent the enrichments of proline and hydroxyproline in product, respectively.

### 2.6. Statistical Analysis

We report measurements as means ± SEM. One-way ANOVA followed by Bonferroni’s multiple comparisons test was performed to test for significant differences between measurements. All statistical analyses were performed using GraphPad Prism 5 (GraphPad Software Inc., San Diego, CA, USA). Tests with *p* < 0.05 were considered significant.

## 3. Results

### 3.1. Intracellular Proline Enrichment in Vastus Lateralis

There were no significant changes in the intracellular enrichment of proline in the vastus lateralis muscle between groups (Table 1). This result indicated that the enrichment of precursor was not affected by exercise or administration.

### 3.2. Mixed Protein Synthesis after Downhill Running Exercise

**Figure 1:**
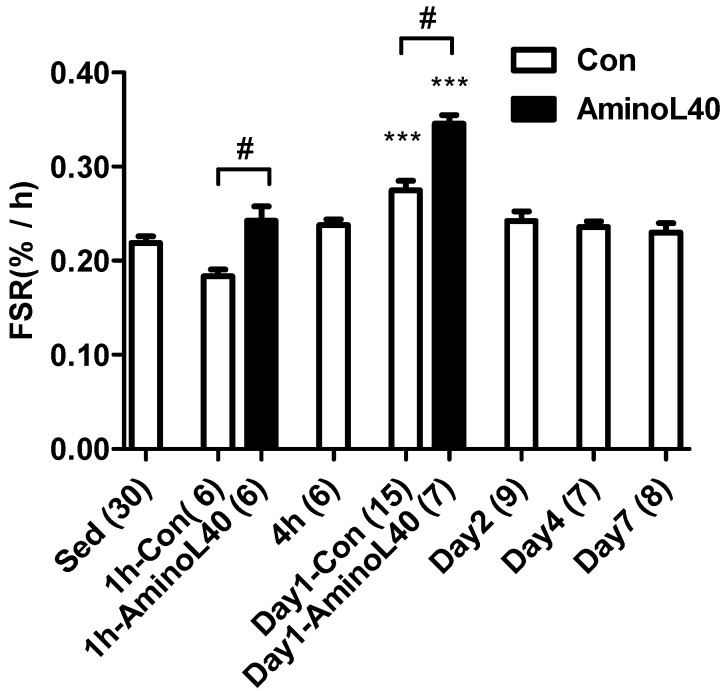
Rates of mixed protein synthesis for the vastus lateralis muscle after downhill running exercise. Mixed protein synthesis was measured in exercised groups with administration of water (open bars) before exercise, 1 h, 4 h, 1 day, 2 days, 4 days, and 7 days, and in exercised groups with administration of LEAA (filled bars) 1 h and 1 day after downhill running exercise. Values are means ± SEM, with n below each bar. #, *p* < 0.05; ***, *p* < 0.001 vs. sedentary group.

Mixed protein synthesis in the vastus lateralis muscle was elevated 1 day after the exercise (Figure 1, *p* < 0.001). LEAA administration led to heightened mixed protein synthesis in the vastus lateralis muscle 1 h and 1 day after the exercise, compared with distilled water administration, at each time point (*p* < 0.05, 0.05, respectively).

### 3.3. Collagen Protein Synthesis after Downhill Running Exercise

**Figure 2:**
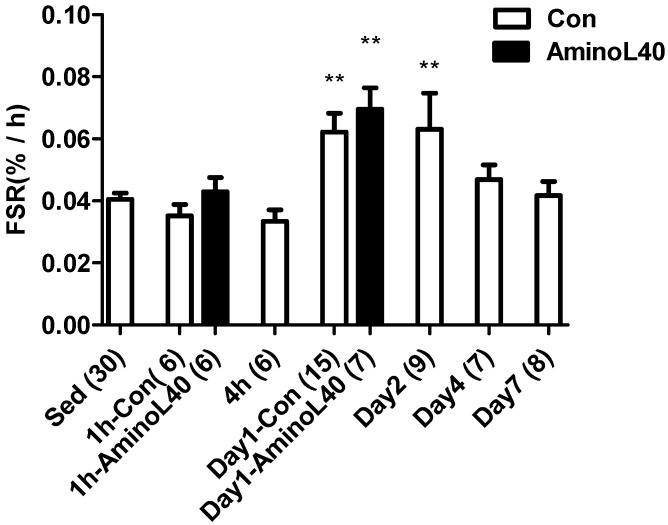
Rates of collagen protein synthesis for the vastus lateralis muscle after downhill running exercise. Collagen protein synthesis was measured in exercised groups with administration of water (open bars) before exercise, 1 h, 4 h, 1 day, 2 days, 4 days, and 7 days, and in exercised groups with administration of LEAA (filled bars) 1 h and 1 day after downhill running exercise. Data are means ± SEM, with n below each bar. **, *p* < 0.01 vs. sedentary group.

Collagen protein synthesis in the vastus lateralis muscle was elevated 1 and 2 days after the exercise (Figure 2, *p* < 0.01, 0.01, respectively), relative to the sedentary group. In contrast to the changes observed for mixed protein synthesis, LEAA administration did not induce a further increase in muscle collagen protein synthesis 1 h or 1 day after the exercise, relative to distilled water administration (*p* > 0.05, 0.05).

### 3.4. Amino Acid Concentrations after Administration of LEAAs Following Downhill Running Exercise

Essential amino acid concentrations in plasma are shown in Table 2. Downhill running exercise did not affect essential amino acid concentrations in plasma 1 h or 1 day after the exercise. At each time point, LEAA administration significantly increased the essential amino acid concentrations by 40%–130%, with the exception of Trp concentrations.

## 4. Discussion

Through measuring the enrichment of hydroxyproline in protein-bound fractions following flooding doses of intravenous d_3_-proline, we found that muscle collagen protein synthesis is elevated and peaks at 1 day after downhill exercise, consistent with reported changes in collagen gene expression [23]. Subsequently, leucine-enriched essential amino acids do not affect muscle collagen protein synthesis 1 h or 1 day after exercise, while the amino acids induce a further increase in mixed muscle protein synthesis 1 day after exercise compared with control. These results are consistent with a previous study on the effects of meal ingestion immediately after exercise [16]. While a peak in muscle collagen protein synthesis presents 1 day after exercise, this process does not respond to enhanced availability of amino acids. As collagen protein synthesis is not affected by nutritional availability at resting state [14,15], muscle collagen protein synthesis might be regulated by mechanical stress rather than nutritional availability.

Nutritional and contractile regulation of global protein synthesis in skeletal muscle is well studied. Exercise increases skeletal muscle protein synthesis by activating the mTOR pathway along with the extracellular signal regulated kinase 1/2 pathway [32]. On the other hand, nutritional availability, particularly of leucine, also leads to skeletal muscle protein synthesis by stimulating the mTOR pathway [33]; such nutritional availability leads to further increases in skeletal muscle protein synthesis after exercise [18,19]. Nutrient and contractile stimuli often converge at mTOR, suggesting that mTOR is an important modulator of protein synthesis. Although molecular signaling activation is not investigated in this study, essential amino acids such as leucine are sufficient to stimulate the mTOR pathway [21,33], and mixed protein synthesis is increased by amino acid intake even in the absence of exercise. We initially hypothesized that mTOR activation would increase muscle collagen protein synthesis, but our observations contradicted this hypothesis. Focusing on the specific regulation of muscle collagen synthesis, the elevated collagen protein synthesis after exercise could be related to mechanical stress by itself and inflammation induced by muscle damage. Mechanical stress induces collagen gene expression in skeletal muscle via integrin [4]. Furthermore, interleukin-6, which is produced following muscle damage [34], induces fibroblast to produce collagen [35]. We have previously reported that a leucine-enriched essential amino acid mixture suppresses the inflammatory response after eccentric exercise [36]. In addition, leucine administration alleviates the accumulation of collagen protein in muscle after cryolesion in rodents [37]. The absence of a difference in muscle collagen protein synthesis between LEAA-administered and non-administered groups may result from this amino acid supplement simultaneously activating the mTOR pathway and suppressed inflammation. Further studies are required to resolve the discrepancy between the activated mTOR pathway and the apparent non-responsiveness of skeletal muscle collagen protein synthesis to nutrient intake.

We found collagen protein synthesis was increased 1 and 2 days after the downhill running, while mixed protein synthesis was increased 1 day, but not 2 days after the exercise. Muscle collagen protein synthesis is increased by mechanical stress [4,38] and inflammation [35]. In fact, 4.5–8.5 h after exercise, increases in collagen protein synthesis are identical for both shortening contractions and lengthening contractions [6]. On the other hand, downhill running in rats, which requires eccentric contraction of the quadriceps, results in more muscle damage than either uphill or level running [39]. For rats, muscle collagen accumulation occurs during the repair process of exercise-induced muscle injuries in rats [40]. Thus, muscle damage-related inflammation seems the most probable explanation for the presently reported increase in muscle collagen protein synthesis. Furthermore, muscle damage-induced inflammation is found starting at 1 day after the exercise, and not sooner [34]. In addition, gene expression of collagen protein was increased 6 hours to 4 days after the downhill running [23,38]. Therefore, beginning 1 day after exercise, muscle damage-induced inflammation may increase muscle collagen protein synthesis. This result applies to the period following eccentric, muscle damage-inducing exercise.

Gene expression for muscle collagen protein is stimulated by mechanical stress [4,38]. Next, procollagen is synthesized in the endoplasmic reticulum, and it undergoes posttranslational modification before being extruded from the endoplasmic reticulum to extracellular space. To the best of our knowledge, the present study is the first to assess muscle collagen protein synthesis measuring hydroxyproline enrichment in the protein-bound fraction after stable isotope labeled-proline injection. This fraction is thought to contain pre-matured collagen (tropocollagen) along with matured collagen molecules. In contrast to previous studies in which the insoluble collagen protein (i.e., matured collagen) is assessed [14,15,16], the present measurements are sensitive to changes in muscle collagen protein. Due to the use of stable isotope-labeled proline, the present study’s method should be applicable for human study to assess collagen protein synthesis measuring the enrichment of hydroxyproline.

## 5. Conclusions

Through measuring the enrichment of hydroxyproline and proline in skeletal muscle, we find that downhill running exercise increases mixed protein synthesis for 1 day following exercise and collagen protein synthesis for 2 days following exercise. At 1 day post-exercise—the peak of post-exercise protein synthesis—administration of leucine-enriched essential amino acids can lead to a further increase in mixed protein synthesis, but not collagen protein synthesis. These results suggest that, contrary to regulation of mixed protein synthesis, muscle collagen protein synthesis is not affected by nutrient availability.

## Figures and Tables

**Table 1:** Intracellular proline enrichment in the vastus lateralis muscle in sedentary (Sed) and exercised groups of rats administered with water (Con) or LEAA (1 g/kg; AminoL40).

	Intracellular Proline Enrichment (%MPE)
Sed (30)	40.5 ± 0.2
1h-Con (6)	42.2 ± 0.2
1h-AminoL40 (6)	42.2 ± 0.3
4h (6)	40.9 ± 0.6
Day1-Con (15)	40.9 ± 0.4
Day1-AminoL40 (7)	39.5 ± 1.0
Day2 (9)	39.4 ± 0.9
Day4 (7)	40.6 ± 0.2
Day7 (8)	39.5 ± 1.0

Data are shown as means ± SEM (Number of rats in each group (n) in parenthesis). No significant differences between groups.

**Table 2:** Plasma essential amino acid concentrations in sedentary (Sed) and exercised (Con) groups of rats administered water (Control) or AminoL40 (1 g LEAA/kg) immediately or 1 day after exercise.

Amino Acid	Sed (14)	Immediately after		1 Day after	
		Con (6)	LEAA (6)	Con (7)	LEAA (8)
His	46.6 ± 2.3 c, d	57.3 ± 2.5 b, c	71.0 ± 3.3 a	39.2 ± 2.1 d	58.2 ± 3.2 b
Ile	71.5 ± 2.1 b	84.8 ± 2.6 b	368.3 ± 25.5 a	62.1 ± 2.8 b	313.5 ± 22.4 a
Leu	115.2 ± 4.2 b	140.5 ± 4.3 b	1448.7 ± 101.3 a	99.9 ± 5.2 b	1304.8 ± 89.2 a
Lys	574.1 ± 23.7 c	630.9 ± 31.2 c	1849.6 ± 91.3 a	529.5 ± 33.2 c	1599.4 ± 47.8 b
Met	58.9 ± 1.7 c	53.1 ± 1.9 c	83.8 ± 4.8 b	50.3 ± 2.2 c	99.5 ± 4.7 a
Phe	59.9 ± 2.1 b	65.3 ± 2.4 b	118.4 ± 7.3 a	53.7 ± 1.7 b	101.4 ± 5.8 a
Thr	232.5 ± 10.4 b	208.2 ± 6.4 b	633.3 ± 70.8 a	223.0 ± 11.6 b	746.7 ± 46.4 a
Trp	98.6 ± 4.5 b, c	116.2 ± 13.0 a, b	145.9 ± 10.0 a	82.6 ± 6.6 c	111.3 ± 4.4 b, c
Val	164.0 ± 6.4 c	195.8 ± 5.4 c	1033.7 ± 50.5 a	145.4 ± 6.2 c	874.8 ± 44.9 b

Data are shown as means ± SEM (Number of rats in each group (n) in parenthesis) in µM. Different letters denote significance of difference, at least *p* < 0.05.

## Abbreviations

The following abbreviations are used in this manuscript:

FSR	fractional synthesis rate
LEAAs	Leucine-enriched essential amino acids
MPE	mole per excess
mTOR	Mammalian target of rapamycin

## References

[1] Huijing P.A. (1999). Muscle as a collagen fiber reinforced composite: A review of force transmission in muscle and whole limb. J. Biomech..

[2] Kovanen V., Suominen H., Heikkinen E. (1980). Connective tissue of “fast” and “slow” skeletal muscle in rats—Effects of endurance training. Acta Physiol. Scand..

[3] Williams P.E., Goldspink G. (1981). Connective tissue changes in surgically overloaded muscle. Cell Tissue Res..

[4] Kjaer M. (2004). Role of extracellular matrix in adaptation of tendon and skeletal muscle to mechanical loading. Physiol. Rev..

[5] Stauber W.T. (2004). Factors involved in strain-induced injury in skeletal muscles and outcomes of prolonged exposures. J. Electromyogr. Kinesiol..

[6] Moore D.R., Phillips S.M., Babraj J.A., Smith K., Rennie M.J. (2005). Myofibrillar and collagen protein synthesis in human skeletal muscle in young men after maximal shortening and lengthening contractions. Am. J. Physiol. Endocrinol. Metab..

[7] Cuthbertson D.J., Babraj J., Smith K., Wilkes E., Fedele M.J., Esser K., Rennie M. (2006). Anabolic signaling and protein synthesis in human skeletal muscle after dynamic shortening or lengthening exercise. Am. J. Physiol. Endocrinol. Metab..

[8] Miller B.F., Olesen J.L., Hansen M., Dossing S., Crameri R.M., Welling R.J., Langberg H., Flyvbjerg A., Kjaer M., Babraj J.A. (2005). Coordinated collagen and muscle protein synthesis in human patella tendon and quadriceps muscle after exercise. J. Physiol..

[9] Phillips S.M., Tipton K.D., Aarsland A., Wolf S.E., Wolfe R.R. (1997). Mixed muscle protein synthesis and breakdown after resistance exercise in humans. Am. J. Physiol..

[10] Biolo G., Maggi S.P., Williams B.D., Tipton K.D., Wolfe R.R. (1995). Increased rates of muscle protein turnover and amino acid transport after resistance exercise in humans. Am. J. Physiol..

[11] Burd N.A., Tang J.E., Moore D.R., Phillips S.M. (2009). Exercise training and protein metabolism: Influences of contraction, protein intake, and sex-based differences. J. Appl. Physiol..

[12] Biolo G., Tipton K.D., Klein S., Wolfe R.R. (1997). An abundant supply of amino acids enhances the metabolic effect of exercise on muscle protein. Am. J. Physiol..

[13] Moore D.R., Robinson M.J., Fry J.L., Tang J.E., Glover E.I., Wilkinson S.B., Prior T., Tarnopolsky M.A., Phillips S.M. (2009). Ingested protein dose response of muscle and albumin protein synthesis after resistance exercise in young men. Am. J. Clin. Nutr..

[14] Babraj J.A., Cuthbertson D.J., Smith K., Langberg H., Miller B., Krogsgaard M.R., Kjaer M., Rennie M.J. (2005). Collagen synthesis in human musculoskeletal tissues and skin. Am. J. Physiol. Endocrinol. Metab..

[15] Mittendorfer B., Andersen J.L., Plomgaard P., Saltin B., Babraj J.A., Smith K., Rennie M.J. (2005). Protein synthesis rates in human muscles: Neither anatomical location nor fibre-type composition are major determinants. J. Physiol..

[16] Holm L., van Hall G., Rose A.J., Miller B.F., Doessing S., Richter E.A., Kjaer M. (2010). Contraction intensity and feeding affect collagen and myofibrillar protein synthesis rates differently in human skeletal muscle. Am. J. Physiol. Endocrinol. Metab..

[17] Katsanos C.S., Kobayashi H., Sheffield-Moore M., Aarsland A., Wolfe R.R. (2006). A high proportion of leucine is required for optimal stimulation of the rate of muscle protein synthesis by essential amino acids in the elderly. Am. J. Physiol. Endocrinol. Metab..

[18] Dreyer H.C., Drummond M.J., Pennings B., Fujita S., Glynn E.L., Chinkes D.L., Dhanani S., Volpi E., Rasmussen B.B. (2008). Leucine-enriched essential amino acid and carbohydrate ingestion following resistance exercise enhances mtor signaling and protein synthesis in human muscle. Am. J. Physiol. Endocrinol. Metab..

[19] Fujita S., Dreyer H.C., Drummond M.J., Glynn E.L., Cadenas J.G., Yoshizawa F., Volpi E., Rasmussen B.B. (2007). Nutrient signalling in the regulation of human muscle protein synthesis. J. Physiol..

[20] Drummond M.J., Rasmussen B.B. (2008). Leucine-enriched nutrients and the regulation of mammalian target of rapamycin signalling and human skeletal muscle protein synthesis. Curr. Opin. Clin. Nutr. Metab. Care.

[21] Kato H., Suzuki H., Mimura M., Inoue Y., Sugita M., Suzuki K., Kobayashi H. (2015). Leucine-enriched essential amino acids attenuate muscle soreness and improve muscle protein synthesis after eccentric contractions in rats. Amino Acids.

[22] Rowlands D.S., Nelson A.R., Raymond F., Metairon S., Mansourian R., Clarke J., Stellingwerff T., Phillips S.M. (2016). Protein-Leucine Ingestion Activates a Regenerative Inflammo-Myogenic Transcriptome in Skeletal Muscle Following Intense Endurance Exercise. Physiol. Genom..

[23] Han X.Y., Wang W., Komulainen J., Koskinen S.O., Kovanen V., Vihko V., Trackman P.C., Takala T.E. (1999). Increased mRNAs for procollagens and key regulating enzymes in rat skeletal muscle following downhill running. Pflügers Archiv.

[24] Witard O.C., Jackman S.R., Breen L., Smith K., Selby A., Tipton K.D. (2014). Myofibrillar muscle protein synthesis rates subsequent to a meal in response to increasing doses of whey protein at rest and after resistance exercise. Am. J. Clin. Nutr..

[25] McAnulty R.J. (2005). Methods for measuring hydroxyproline and estimating in vivo rates of collagen synthesis and degradation. Methods Mol. Med..

[26] Laurent G.J. (1987). Dynamic state of collagen: Pathways of collagen degradation in vivo and their possible role in regulation of collagen mass. Am. J. Physiol..

[27] McAnulty R.J., Laurent G.J. (1987). Collagen synthesis and degradation in vivo. Evidence for rapid rates of collagen turnover with extensive degradation of newly synthesized collagen in tissues of the adult rat. Coll. Relat. Res..

[28] Laurent G.J. (1982). Rates of collagen synthesis in lung, skin and muscle obtained in vivo by a simplified method using [3H] proline. Biochem. J..

[29] Smith K., Rennie M.J. (2007). New approaches and recent results concerning human-tissue collagen synthesis. Curr. Opin. Clin. Nutr. Metab. Care.

[30] Dietzen D.J., Weindel A.L., Carayannopoulos M.O., Landt M., Normansell E.T., Reimschisel T.E., Smith C.H. (2008). Rapid comprehensive amino acid analysis by liquid chromatography/tandem mass spectrometry: Comparison to cation exchange with post-column ninhydrin detection. Rapid Commun. Mass Spectrom..

[31] Calder A.G., Anderson S.E., Grant I., McNurlan M.A., Garlick P.J. (1992). The determination of low d5-phenylalanine enrichment (0.002–0.09 atom percent excess), after conversion to phenylethylamine, in relation to protein turnover studies by gas chromatography/electron ionization mass spectrometry. Rapid Commun. Mass Spectrom..

[32] Drummond M.J., Dreyer H.C., Fry C.S., Glynn E.L., Rasmussen B.B. (2009). Nutritional and contractile regulation of human skeletal muscle protein synthesis and mtorc1 signaling. J. Appl. Physiol..

[33] Crozier S.J., Kimball S.R., Emmert S.W., Anthony J.C., Jefferson L.S. (2005). Oral leucine administration stimulates protein synthesis in rat skeletal muscle. J. Nutr..

[34] Peake J., Nosaka K., Suzuki K. (2005). Characterization of inflammatory responses to eccentric exercise in humans. Exerc. Immunol. Rev..

[35] Duncan M.R., Berman B. (1991). Stimulation of collagen and glycosaminoglycan production in cultured human adult dermal fibroblasts by recombinant human interleukin 6. J. Investig. Dermatol..

[36] Kato H., Miura K., Nakano S., Suzuki K., Bannai M., Inoue Y. (2016). Leucine-enriched essential amino acids attenuate inflammation in rat muscle and enhance muscle repair after eccentric contraction. Amino Acids.

[37] Pereira M.G., Silva M.T., Carlassara E.O., Goncalves D.A., Abrahamsohn P.A., Kettelhut I.C., Moriscot A.S., Aoki M.S., Miyabara E.H. (2014). Leucine supplementation accelerates connective tissue repair of injured tibialis anterior muscle. Nutrients.

[38] Koskinen S.O., Wang W., Ahtikoski A.M., Kjaer M., Han X.Y., Komulainen J., Kovanen V., Takala T.E. (2001). Acute exercise induced changes in rat skeletal muscle mRNAs and proteins regulating type IV collagen content. Am. J. Physiol. Regul. Integr. Comp. Physiol..

[39] Schwane J.A., Armstrong R.B. (1983). Effect of training on skeletal muscle injury from downhill running in rats. J. Appl. Physiol. Respir. Environ. Exerc. Physiol..

[40] Myllyla R., Salminen A., Peltonen L., Takala T.E., Vihko V. (1986). Collagen metabolism of mouse skeletal muscle during the repair of exercise injuries. Pflügers Archiv.

